# Elucidating potential molecular signatures through host-microbe interactions for reactive arthritis and inflammatory bowel disease using combinatorial approach

**DOI:** 10.1038/s41598-020-71674-8

**Published:** 2020-09-15

**Authors:** Anukriti Verma, Shivani Sharda, Bhawna Rathi, Pallavi Somvanshi, Bimlesh Dhar Pandey

**Affiliations:** 1 Amity Institute of Biotechnology, J-3 Block, Amity University Campus, Sector-125, Noida, UP 201313 India; 2 Department of Biotechnology, TERI School of Advanced Studies, 10, Institutional Area, Vasant Kunj, New Delhi, 110070 India; 3 Fortis Hospital, B-22, Sector 62, Gautam Buddh Nagar, Noida, Uttar Pradesh 201301 India

**Keywords:** Computational biology and bioinformatics, Biomarkers

## Abstract

Reactive Arthritis (ReA), a rare seronegative inflammatory arthritis, lacks exquisite classification under rheumatic autoimmunity. ReA is solely established using differential clinical diagnosis of the patient cohorts, where pathogenic triggers linked to enteric and urogenital microorganisms e.g. *Salmonella*, *Shigella*, *Yersinia*, *Campylobacter*, *Chlamydia* have been reported. Inflammatory Bowel Disease (IBD), an idiopathic enteric disorder co-evolved and attuned to present gut microbiome dysbiosis, can be correlated to the genesis of enteropathic arthropathies like ReA. Gut microbes symbolically modulate immune system homeostasis and are elementary for varied disease patterns in autoimmune disorders. The gut-microbiota axis structured on the core host-microbe interactions execute an imperative role in discerning the etiopathogenesis of ReA and IBD. This study predicts the molecular signatures for ReA with co-evolved IBD through the enveloped host-microbe interactions and microbe-microbe ‘interspecies communication’, using synonymous gene expression data for selective microbes. We have utilized a combinatorial approach that have concomitant in-silico work-pipeline and experimental validation to corroborate the findings. In-silico analysis involving text mining, metabolic network reconstruction, simulation, filtering, host-microbe interaction, docking and molecular mimicry studies results in robust drug target/s and biomarker/s for co-evolved IBD and ReA. Cross validation of the target/s or biomarker/s was done by targeted gene expression analysis following a non-probabilistic convenience sampling. Studies were performed to substantiate the host-microbe disease network consisting of protein-marker-symptom/disease-pathway-drug associations resulting in possible identification of vital drug targets, biomarkers, pathways and inhibitors for IBD and ReA.

Our study identified Na^(+)^/H^(+)^ anti-porter (NHAA) and Kynureninase (KYNU) to be robust early and essential host-microbe interacting targets for IBD co-evolved ReA. Other vital host-microbe interacting genes, proteins, pathways and drugs include Adenosine Deaminase (ADA), Superoxide Dismutase 2 (SOD2), Catalase (CAT), Angiotensin I Converting Enzyme (ACE), carbon metabolism (folate biosynthesis) and methotrexate. These can serve as potential prognostic/theranostic biomarkers and signatures that can be extrapolated to stratify ReA and related autoimmunity patient cohorts for further pilot studies.

## Introduction

Enteropathic arthropathies symbolize the inflammation of the gut and the joint, where Reactive Arthritis (ReA) and Inflammatory Bowel Disease (IBD) associated arthropathies are caused by bacterial and parasitic infections of the gastrointestinal and urogenital tract. The grouped field of seronegative spondyloarthropathies show involvement of spine, peripheral or lower joints along with extra-articular inflammatory manifestations (beyond joints), with shared characteristics involving the modulations at the gut-joint axis leading to co-evolved autoimmune disorders. ReA is a rare seronegative post-infectious (gastrointestinal or genitourinary) autoimmune disorder, with symptomatic features and concordant dysbiosis, that are shared with seronegative arthropathies such as Ankylosing Spondylitis (AS) and Psoriatic Arthritis (PsA)^1–3^. The prevalence of IBD, an idiopathic inflammatory autoimmunity is not rare with disease progression having shared characteristics of infectious diarrhea and inflammation with seronegative arthropathies. IBD is categorised into Ulcerative Colitis (UC) and Crohn’s Disease (CD) affecting the gastrointestinal tract structured mostly due to gut dysbiosis^4,5^. IBD, clinically associated with AS and PsA, is also voiced to be co-regulated with other forms of seronegative rheumatological associations or spondyloarthropathies^6,7^.

Host genetic histocompatibility molecule HLA (HLA-B27, HLA-B51 and HLA-DRB1), responsible for the regulation of immune system has been reported to be associated with the co-evolved autoimmune dysbiosis (both ReA and IBD)^8,9^. The post-dysenteric ReA is speculated to confer mechanism of molecular mimicry (sequence cross reactivity of gut derived microbial antigen with host HLA protein) leading to autoantibody development, inflammatory response and tissue damage in host^10–12^. The exact detailing of the process however is not confirmed in either clinical setting or in experimental colitis models^12–14^. The segregated contribution of HLA-B27 related genetic alleles in aggravating pathogenesis in ReA patients, confer inconclusive significance of these genes as the singular biomarker due to the varied clinical patterns in patient cohorts.

Further, the immune triggering in such microbial rheumatism is due to the disturbance of the gut barrier, disruptions in immune regulatory mechanisms, translocation of pathogens or microbial elements, trafficking of gut derived immunocompetent cells, immune response complexes as well as metabolites.

Thus, the co-evolved inflammatory manifestations in IBD and ReA show the cumulative contribution of HLA-B27, microbes, proinflammatory cytokines (TLR’s e.g. TLR-4) and immunogenic factors like CRP, IL-6, IL-13, IFN-γ and TNF in the Th2 and Th1 mediated responses in the pathogenicity of ReA and IBD^15–18^.

The autoimmune disorders are studied mostly through genetic, environmental and immunogenic features using direct or indirect (animal models) estimation studies that predispose individuals to gut-joint inflammatory conditions. Consequently, etiopathogenesis mechanisms for each disorder have been derived separately, though the concurring triggering elements for precise pathogenic modulation of such disorders having shared characteristics have not been coordinated. Shifts in gut microbiome composition occur when the commensal gut microbes (symbiotic relationships with human host confining inflammatory response and regulating adaptive and innate immunity) shift towards dysbiosis caused via pathogenic microbes contributing in the immuno-pathogenesis of enteropathic disorders, IBD and ReA^19^.

Clinical variables and chronic enteric infection exemplars, analysed from the patient cohorts have ascertained the contribution of various species of *Salmonella*, *Shigella*, *Yersinia* and *Campylobacter* enteropathogens along with *Chlamydia* as the co-occuring taxa at the host–pathogen interface having precedence in both ReA and IBD^3,20–25^. The infectious regime in both ReA and IBD patient cohorts have been followed after episode of gastroenteritis through either salmonellosis, shigellosis, yersiniosis, campylobacteriosis or enterocoloitis^3,20,23,26,27^ or urogenital infections through microbial species of *Chlamydia*^21,22^, where either the microbe itself, specific antibodies or their genetic components have been reported in the joint fluid following infection.

The post-infectious development and dysregulation due to the microbes or their associated metabolites could serve as potential arthritogenic targets in post-enteric arthritis, where immunological cross-reactivity leads to generation of auto-antibodyome^28,29^. This is due to presence of microbe encoded peptides, outer membrane proteins, microbial metabolites or antigens found in synovial fluid, serum and fecal samples of patients^1,13,14,30–36^^.^ Thus, the metabolite analyses could systematically validate contribution of microorganisms and microbial bioactive molecules stimulating diverse pathophysiological signaling pathways as the generic triggers for co-evolved disorders^37–39^.

The management of rare complex overlapping autoimmune disorder ReA so as to prevent its progression and recurrence^40–42^ depends on the detailed microbiome association studies with the host genetics structuring the immunogenic trends^43–45^. The gut derived core host-microbe and microbe-microbe ‘interspecies communication’ would enhance the existing knowledge of such co-existent disorders IBD and ReA, while similar approach has recently been followed by Manasson et al., 2018 for ReA and PsA. Due to the dearth in the experimentally derived analyses of rare disorder ReA, expression profiling of co-evolved IBD with detailed information of the molecular signatures were estimated that can postulate new therapeutic tools and disease prevention strategies for ReA.

The available data can unlock the potential common targets in the enteropathic autoimmunity by evaluation of components at multi-omic levels (genomics, transcriptomics, proteomics, metabolomics and interactomics)^46^ for the common triggering microbes that can unravel many interesting biological associations and events associated with the infectious disorders IBD and ReA.

The combinatorial study pipeline was designed to utilize in-silico systems biology approaches using metabolic network reconstructions for analyzing biochemical transformations to comprehend the significant interplay of gut-microbiome modulations between myriad of molecules such as genes, proteins, metabolites or biomarkers and varying pathways as previously documented^47,48^. Simulation studies for gene essentiality analysis, gene filtration by removing the paralogs and homologs, network analysis to understand the function of proteins, association between protein-pathway, disease-gene, disease-disease and protein–protein interaction networks studies were inculcated to find more robust targets and hub proteins^49,50^. Further, gene expression data thus helps predicts the immunopathogenesis involving the common microbes and microbial components either singularly or through the closely-knit connections between contributing microorganisms to postulate the synthesis of post-infectious ReA and IBD.

We focus on multi-network analysis incorporating the simultaneous associations from the selected microorganisms collectively while most of these associations have been studied individually in other studies. This new approach has advantages over the traditional approach for network analysis that can help to simultaneously characterize several protein interaction modules and has the potential to study complex diseases. The vital information obtained in our study from in-silico analysis is cross-validated through targeted gene expression experimental analysis on patient cohorts. This study will help us to obtain clinico-molecular informatics-based outcomes and expand our knowledge regarding the understanding of biological functions for IBD co-existent ReA.

## Materials and methods

### Text mining: data screening and selection

Systematic data search and organization was carried out incorporating data identification, data screening and data selection to find target microorganisms involved in Inflammatory Bowel Disease (IBD) and Reactive Arthritis (ReA). Data identification was carried out to obtain records through data sources utilising keywords (e.g. “Microorganism AND Inflammatory bowel disease AND Reactive arthritis”) incorporating Boolean operators (AND/OR/NOT). Data screening and selection were carried as part of the manual curation through primary and secondary screening scrutinizing collected data records to obtain organized records relevant for the autoimmune and enteric disorders triggered by microorganisms, especially IBD and ReA and the microbial triggers implicated in IBD and ReA that were utilised for further metabolic network reconstruction.

### Metabolic network reconstruction, simulation analysis and data filtering

A constrained based bottom-up approach consisting of draft reconstruction and manual reconstruction refinement was followed to create metabolic networks of obtained target microorganisms. Genome-scale Metabolic models Simulation, Reconstruction and Visualization (GEMSiRV) software^51^ that includes reciprocal Basic Local Alignment Search Tool (BLAST) of target microorganisms against a template metabolic network of its phylogenetic neighbour and incorporates information from National Center for Biotechnology Information (NCBI), Kyoto Encyclopedia of Genes and Genomes (KEGG) and Transport DB was used for creating draft reconstructs. The manual curation of missing links or gaps in the draft reconstruct was done by mapping the incomplete information to other databases such as Expert Protein Analysis System (ExPASy)^52^ and Integrated relational Enzyme database (IntEnz)^53^. This fully connected and annotated network was used for further simulation studies^54^. The metabolic networks thus obtained were visualized using CellDesigner, a tool for modelling and editing biochemical and gene-regulatory networks.

Simulation analysis was carried by converting the metabolic networks obtained into a mathematical model and performing the gene deletion analysis to retrieve essential genes. Model conversion was through generation of stoichiometric based matrixes consisting of reactions (columns) and metabolites (rows) corresponding to respective genes. Upper boundary and lower boundary fluxes i.e. movement of matter across a system were generated for the gene associated reactions and metabolites that was extracted in Systems Biology Markup Language (SBML) format. The next step was gene deletion analysis done using the Constraint Based Reconstruction and Analysis toolbox (COBRA) that runs in Matrix Laboratory (MATLAB)^55^ for finding the essential genes based upon the gene-reaction matrix and boolean relationship between genes and reactions^56^.

The purpose of data filtering is to remove repeats and homologs from essential genes of target microorganisms associated with IBD co-existent ReA. The non-homologous protein sequences corresponding to the essential genes of target microorganisms were extracted from Pathosystems Resource Integration (PATRIC) database^57^. Refinement of protein sequences was further done using Cluster Database at High Identity with Tolerance (CD-HIT)^58^ suite so as to have 60% identity non-repeat sequence tolerance stringency. BLAST-P was further used to remove the homologs from such non-repeats against human database at e-value of 10^–4^ to obtain non-homologous protein sequences used for further in-silico analysis.

### Essential host-microbe and microbe-microbe interactions

The host-microbe interactions of the non-homologous proteins for the selected target microorganisms were obtained using Host–pathogen Interaction Database (HPIDB)^59,60^. The host-microbe interactions were visualised using Cytoscape. Simulation analysis (gene essentiality) was done to obtain the essential host proteins interacting with common microbe proteins of microorganisms triggering IBD and ReA utilising the human metabolic model HMR 2, a COBRA compliant metabolic model of human consisting of around 3,765 genes, 8,000 reactions and 3,000 metabolites^61^.

This led to profiling of the common host-microbe and microbe-microbe interactions comprehending the complex ‘interspecies communication’ as complex interaction maps, executed using Search Tool for the Retrieval of Interacting Genes/proteins (STRING)^62,63^.

### Host-microbe disease network and molecular mimicry studies

The host-microbe disease network is a multilayered archetype that connects the protein-marker-symptom/disease-drug-pathway associations. The contributions of the microorganisms in the co-evolved IBD and ReA as part of the disease network was created through the interactive maps of the essential host interaction proteins (verified using literature survey) and the information processed through gene expression data analysis^64^. The information patronised here is mostly scored through the available non-specific protein diagnostic markers of both IBD and ReA e.g. C-Reactive Protein (CRP), Interleukin 6 (IL6) and Toll Like Receptor 4 (TLR4), Major Histocompatibility Complex, Class I, B (HLA-B) and Major Histocompatibility Complex, Class II, DR Beta 1 (HLA-DRB1) with the essential host proteins determined using STRING^65^.

Database GeneCards^66^ was used to assess the role of these interacting partners aka proteins further with symptoms/diseases associated with IBD and ReA. The pathways of the above host interacting proteins were found out using KEGG database that provides ontologies for proteins related to biological processes^67^.

Subsequently, the role of drugs or inhibitors used to suppress the effect of IBD and ReA such as indomethacin, prednisone, ciprofloxacin, sulfasalazine, azathioprine, methotrexate and hydroxychloroquine was scored in the disease network through their docking studies against the potential targets (both host as well microbial targets) as per published methodologies^68,69^. The host-microbe disease network which is an amalgamation of all the above patterned associations was visualized using Cytoscape software^70^.

Molecular mimicry analysis between the vital targets triggering IBD co-evolved ReA, essential human proteins including HLA-B27, HLA-B51 and HLA-DRB1 was done using data repository ExPASy. This led to retrieval of microbe relayed protein sequences that have been implicated in disease development after sequence alignment performed using EMBOSS^71^.

### Experimental evidences to identify the signature molecules in patient samples

The cross-validation of vital in-silico targets was done in ReA patient cohort cases via targeted gene expression analysis. Scientific and ethical clearance was taken from Amity University Ethics Committee and Institutional Ethics Committee, Fortis Noida for handling the patient samples. All experiments were performed in accordance with Indian Council of Medical Research (ICMR) guidelines constituting the ethics committees. The study was carried out for 6 months on the rare disorder ReA patients, with the inclusion criteria as patients having ReA according to European Spondyloarthropathy Study Group (ESSG)^72^ and exclusion criteria as patients undergoing treatment from last 3–6 months and healthy controls (HC).

The participants were inducted in the study design with an informed consent form along with a questionnaire containing information regarding symptomatic and diagnostic history of patient and linked disorders.

Blood (5 mL) was drawn from participants in ethylenediaminetetraacetic acid (EDTA) vacutainers. These were transported to the laboratory for further analysis. The processing of the samples was done within 2–4 h of procurement^73^. Peripheral blood mononuclear cells (PBMC’s) were isolated from blood using density gradient centrifugation^74^. RNA was isolated from PBMC’s using TRIzol method^75^. The quantification of RNA was done using nano-drop^76^. The High Capacity cDNA Reverse Transcription Kit (Applied Biosystems™) was used for conversion of RNA to single-stranded cDNA as per the standard protocol^77^. Quantitative PCR analysis of target gene was executed using Biorad CFX96 Real time-PCR taking human housekeeping gene, GAPDH as a reference. Previously reported primers for qPCR analysis of target and reference gene were selected for this study^78,79^ following the standard protocol^80^. Relative gene expression analysis from qPCR data was performed using the Relative Expression Software Tool (REST® 2009)^81^ that utilises the expression of reference genes to normalize expression of target genes in different samples.

**Figure 1:**
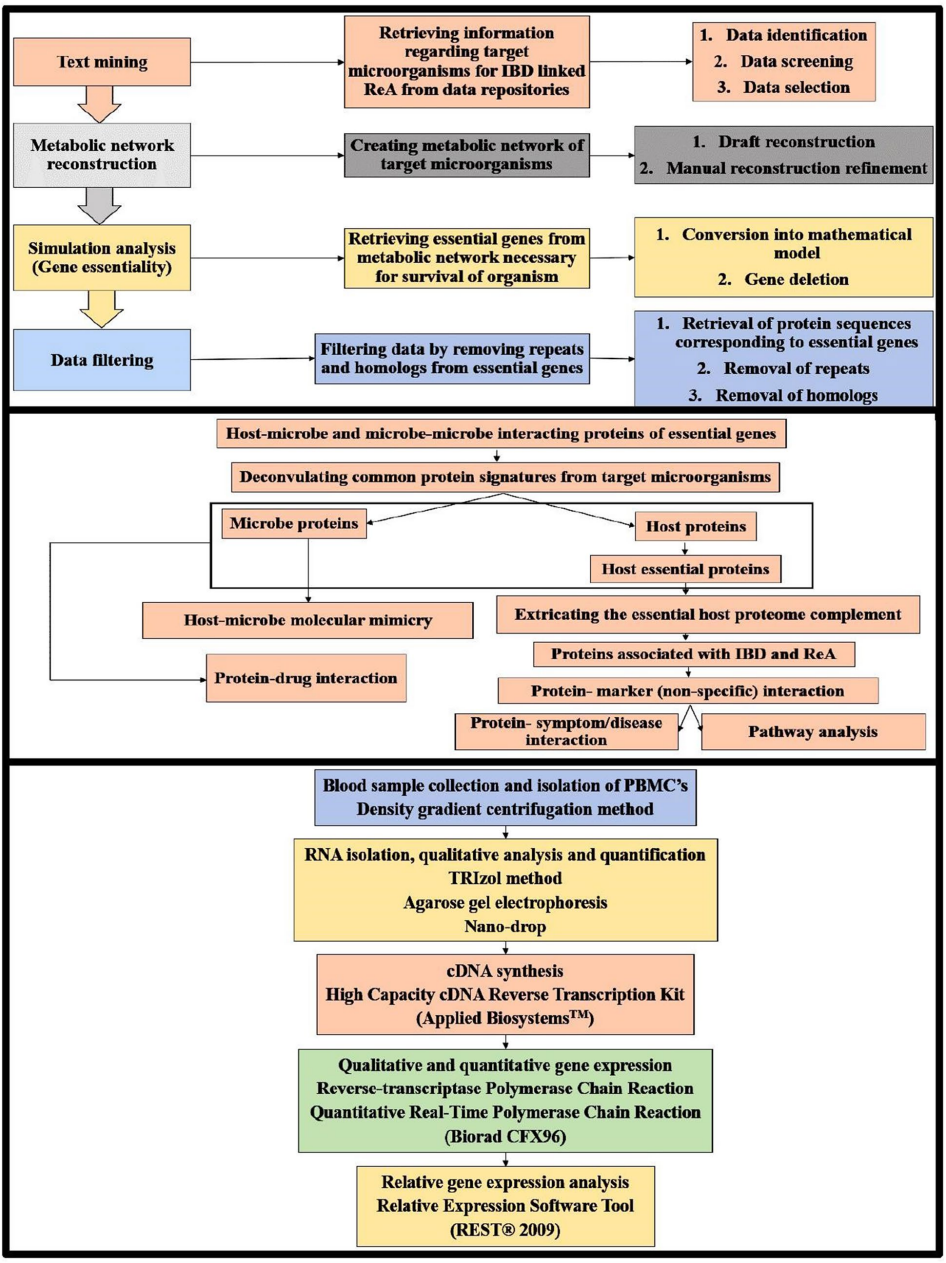
Methodology used for combinatorial analysis.

The schematic representation of methodology involved in our combinatorial analysis is provided in Fig. 1.

## Results

### Text mining: data screening and selection

A systematic literature mining and curation for our thematic connecting autoimmune disorders, Inflammatory Bowel Disease (IBD) and Reactive Arthritis (ReA) was carried out. Data identification extracted 1,071 records (articles in journals, book chapters, conference papers etc.) corresponding to autoimmune and enteric disorders. Data screening extracted 426 records of autoimmune and enteric disorders triggered by microorganisms that belong to class of bacteria, fungi, protozoan, mites, virus, yeast and nematode. Data selection yielded 48 IBD, 32 ReA and 5 IBD co-evolved ReA records. Data selection was directed towards the microbial contenders implicated here resulting in 6 target microorganisms namely *Campylobacter jejuni*, *Escherichia coli O157:H7*, *Klebsiella oxytoca*, *Salmonella typhimurium*, *Shigella dysenteriae and Yersinia enterocolitica*, whose genome information was available. The etiopathogenesis in the co-evolved disorders have been documented through gut microbiome associated host–pathogen interactions studies, perpetuating where pathogen microorganisms involve in dysbiosis leading to autoimmunity.

**Figure 2:**
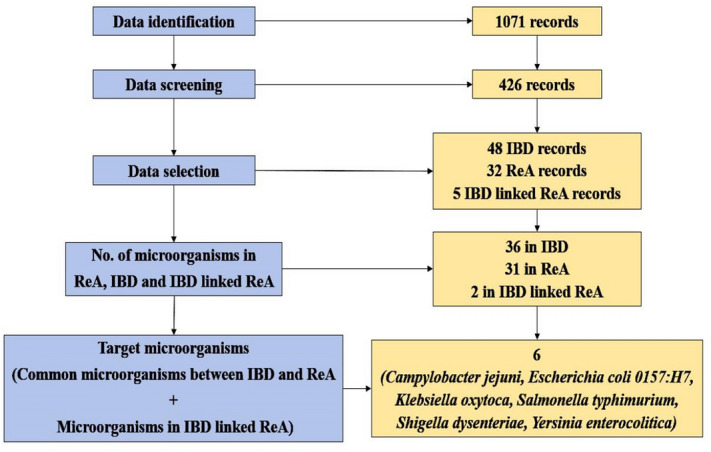
Target microorganisms for IBD co-evolved ReA obtained after text mining.

The results of text mining are provided in Fig. 2. The list of microorganisms is provided in Supplementary Table S1 online.

### Metabolic network reconstruction, simulation and data filtering

The draft reconstructs consisting of genes along with their corresponding proteins, reactions and metabolites for the selected microorganisms serve as primary set of partial metabolic network information. The missing data persistent in the draft reconstruct obtained through Genome-scale Metabolic models Simulation, Reconstruction and Visualization (GEMSiRV) was manually refined. Entirely associated metabolic networks of target microorganisms were obtained (genes, proteins and reactions).

The essential genes of microorganisms (vital for survival. sustenance and growth) were obtained after performing simulation on mathematical models consisting of gene associated reactions and metabolites (metabolites, inner cell reactions, exchange reactions and essential genes).

Due to lack of availability of exchange reactions for *Campylobacter jejuni*, simulation analysis on the partial metabolic network could not be carried out and essential genes could not be retrieved. An alternative approach for finding essential genes of *Campylobacter jejuni* was carried out. The essential genes of *Campylobacter jejuni* were taken from our previous published report and were found out to be 228^69^.

Table 1 portrays the results of metabolic network reconstruction and simulation of target microorganisms. The metabolic network and simulation analysis data of target microorganisms is provided in Supplementary Table S2 online.

**Table 1:** Metabolic network reconstruction (GEMSiRV) and simulation (COBRA) of target microorganisms.

Microorganisms	Genes	Proteins	Reactions	Metabolites	Inner cell reactions	Exchange reactions	Essential genes
*Escherichia coli O157:H7*	1,156	1,057	1824	1,001	1824	283	857
*Klebsiella oxytoca*	1,073	1,001	1794	984	1794	283	833
*Salmonella typhimurium*	1,062	974	1779	980	1779	282	834
*Shigella dysenteriae*	955	892	1623	966	1623	277	735
*Yersinia enterocolitica*	882	821	1573	942	1573	270	726
*Campylobacter jejuni*	45	44	79	140	79	N/A	? (Partial info)

The proteins corresponding to essential genes, non-repeats and non-homologs were obtained as stated below according to the parenthesis {proteins corresponding to essential genes, non-repeats, non-homologs}.

*Escherichia coli O157:H7* {2024, 858, 454}; *Klebsiella oxytoca* {833, 830, 430}; *Salmonella typhimurium* {849, 828, 439}; *Shigella dysenteriae* {735, 732, 382}; *Yersinia enterocolitica* {726, 723, 365} and *Campylobacter jejuni* {153, 153, 151}.

The essential genes, their corresponding proteins, reactions and metabolites from the curated dataset were refined to create a list of most relevant molecular indicators to assess their coveted role in disease establishment. The non-redundant filtered proteins were utilised further in the computational work-pipeline canvassing the drug targets and signatures in the interspecies communication.

### Essential host-microbe and microbe-microbe interactions

The central mechanism of host-microbe/microbe interface conferred through gut microbiome was correlated for the selected microbial species and processed to obtain the common signatures so as to follow the core system of metabolic changes affecting the host harbouring them as either commensal or pathogenic loads. The interactors between human and target microorganisms were obtained.

The interactors of *Escherichia coli O157:H7* were 136; *Klebsiella oxytoca* were 141; *Salmonella typhimurium* were 136; *Shigella dysenteriae* were 117 and *Yersinia enterocolitica* were 133. There were no interactors for *Campylobacter jejuni* (Supplementary Table S3–S7 online). Table 2 shows the results of filtering and host-microbe interactions of protein sequences corresponding to essential genes of target microorganisms.

**Table 2:** Filtering and host-microbe interactions of protein sequences corresponding to essential genes of target microorganisms.

Microorganisms	Corresponding proteins to essential genes	Non-repeats	Non-homologs	Host-microbe interactors
*Escherichia coli O157:H7*	2024	858	454	136
*Klebsiella oxytoca*	833	830	430	141
*Salmonella typhimurium*	849	828	439	136
*Shigella dysenteriae*	735	732	382	117
*Yersinia enterocolitica*	726	723	365	133
*Campylobacter jejuni*	153	153	151	N/A

The host-microbe interactors were analysed for all the target microbial species and processed to obtain the common signatures. 43 proteins were found between all target microorganisms having interaction among themselves and with 130 human proteins.

**Figure 3:**
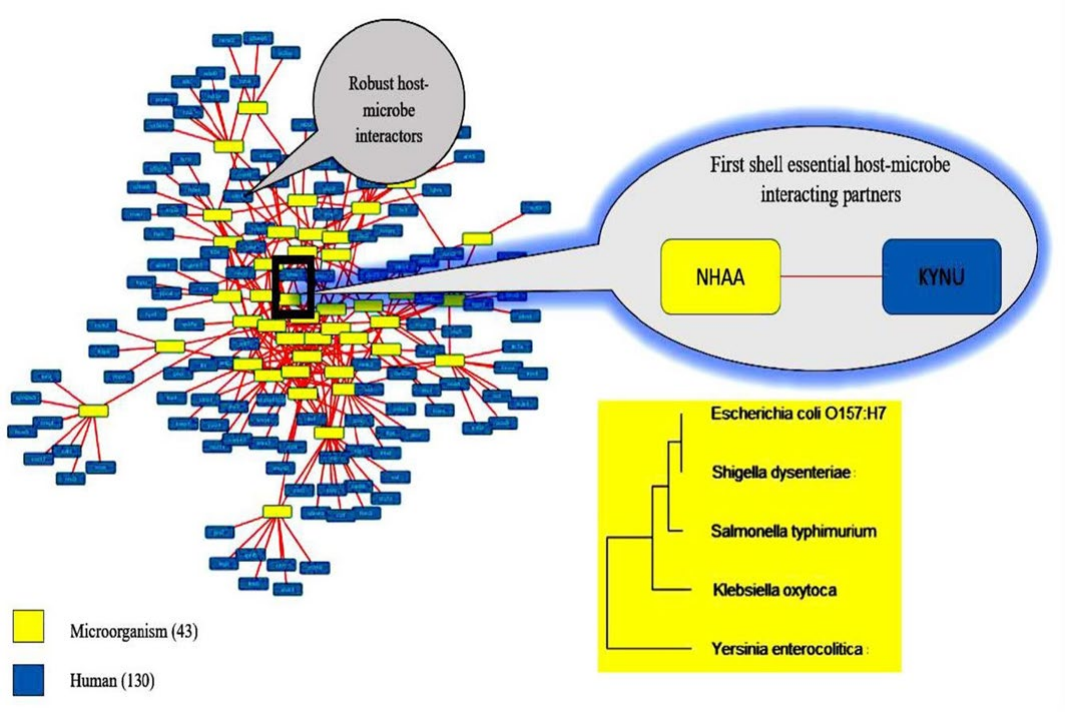
Essential host-microbe interacting proteins.

The essential host correlative targets to the microbial gene targets were followed by obtaining host essential genes and corresponding proteins from human metabolic model HMR 2. There were 1,401 essential proteins (Supplementary Table S8 online) the essential human protein was found out to be KYNU having interaction with essential microbial protein NHAA (Fig. 3). NHAA was also having interactions with non-essential HCLS1 Associated Protein X-1 (HAX1), Prolyl endopeptidase-like (PPCEL), Biogenesis of Lysosomal Organelles Complex 3 Subunit 1 (HPS1) and Eukaryotic Translation Initiation Factor 2 Alpha Kinase 1 (E2AK1) proteins of human host.

**Figure 4:**
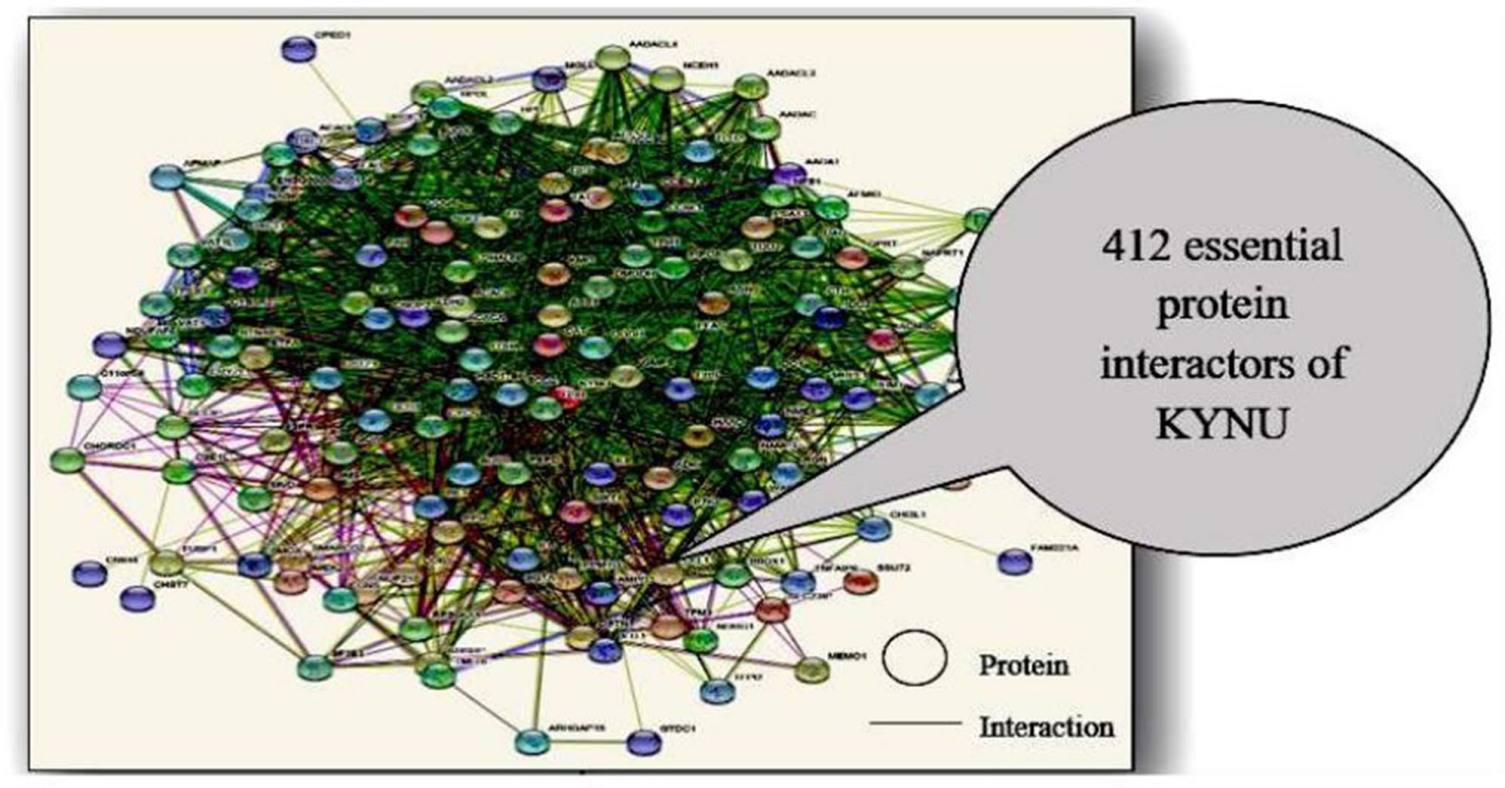
Essential protein interactors of KYNU.

KYNU was further mapped with host proteins (direct and indirect) resulting in 1994 interactions. Out of these the single connected essential protein interactions were 988 and protein interactors were 412 (Fig. 4 and see Supplementary Table S9 online). The research design here followed to assess the interaction map of essential proteins in human host to indicate the clinical insights in pathophysiological trends in the autoimmune development.

### Host-microbe disease network and molecular mimicry

The human essential proteome complement with its interacting proteins were analysed further as part of the disease network. 394 human essential protein interactors were found to be associated with IBD and similarly 3 essential protein interactors namely Adenosine Deaminase (ADA), Catalase (CAT) and Superoxide Dismutase 2 (SOD2) with both IBD and ReA from analysed datasets, published datasets and literature sources (see Supplementary Table S10 online). These 397 proteins can be postulated as probable contenders transcending their role in the simulated network as important regulators in the co-existent disorders.

The composite associations of the above 397 proteins with non-specific protein diagnostic markers of IBD and ReA were obtained (see Supplementary Table S11 online). This gave rise to a single connected protein network consisting of 402 proteins and 13,350 interactions. The association of above 402 with symptoms and diseases linked with IBD and ReA were obtained (see Supplementary Table S12 online). Apart from non-specific diagnostic markers, the major protein linked with majority of symptoms/diseases is Angiotensin I Converting Enzyme (ACE).

78 pathways of the 402 proteins were obtained (see Supplementary Table S13 online) in total out of which the pathway associated with majority of proteins was carbon metabolism.

Another layer of disease network substantiates the role of therapeutic regime followed in the studied autoimmune diseases, so the docking analysis of drugs used to suppress the effect of IBD and ReA against NHAA of target microorganisms and KYNU of human host was done.

The docking analysis resulted in docking scores that represent binding of drugs with host KYNU and microbial NHAA of all 5 microorganisms selected in our study. Higher the negative docking score more is the binding^68^.

*Escherichia coli* *O157:H7* NHAA shows highest and lowest docking score with methotrexate (− 7.362) and azathioprine (− 3.491); *Klebsiella oxytoca* NHAA with methotrexate (− 5.083) and azathioprine (− 3.459); *Salmonella typhimurium* NHAA with ciprofloxacin (− 5.135) and hydroxychloroquine (− 2.597); *Shigella dysenteriae* NHAA with methotrexate (− 8.059) and azathioprine (− 3.847); *Yersinia enterocolitica* NHAA with hydroxychloroquine (− 7.47) and azathioprine (− 3.451) and human KYNU with hydroxychloroquine (− 5.357) and indomethacin (1.113). Our results portray methotrexate to have highest docking scores with maximum proteins and therefore can be considered as a vital drug for IBD associated ReA.

**Figure 5:**
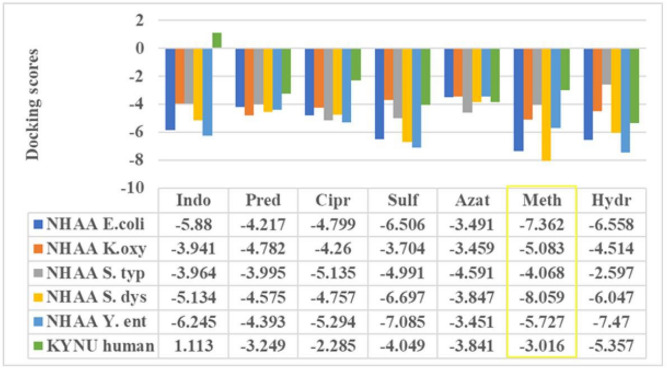
Docking scores of drugs utilised in IBD and ReA against NHAA and KYNU.

The resultant docking scores are provided in Fig. 5.

**Figure 6:**
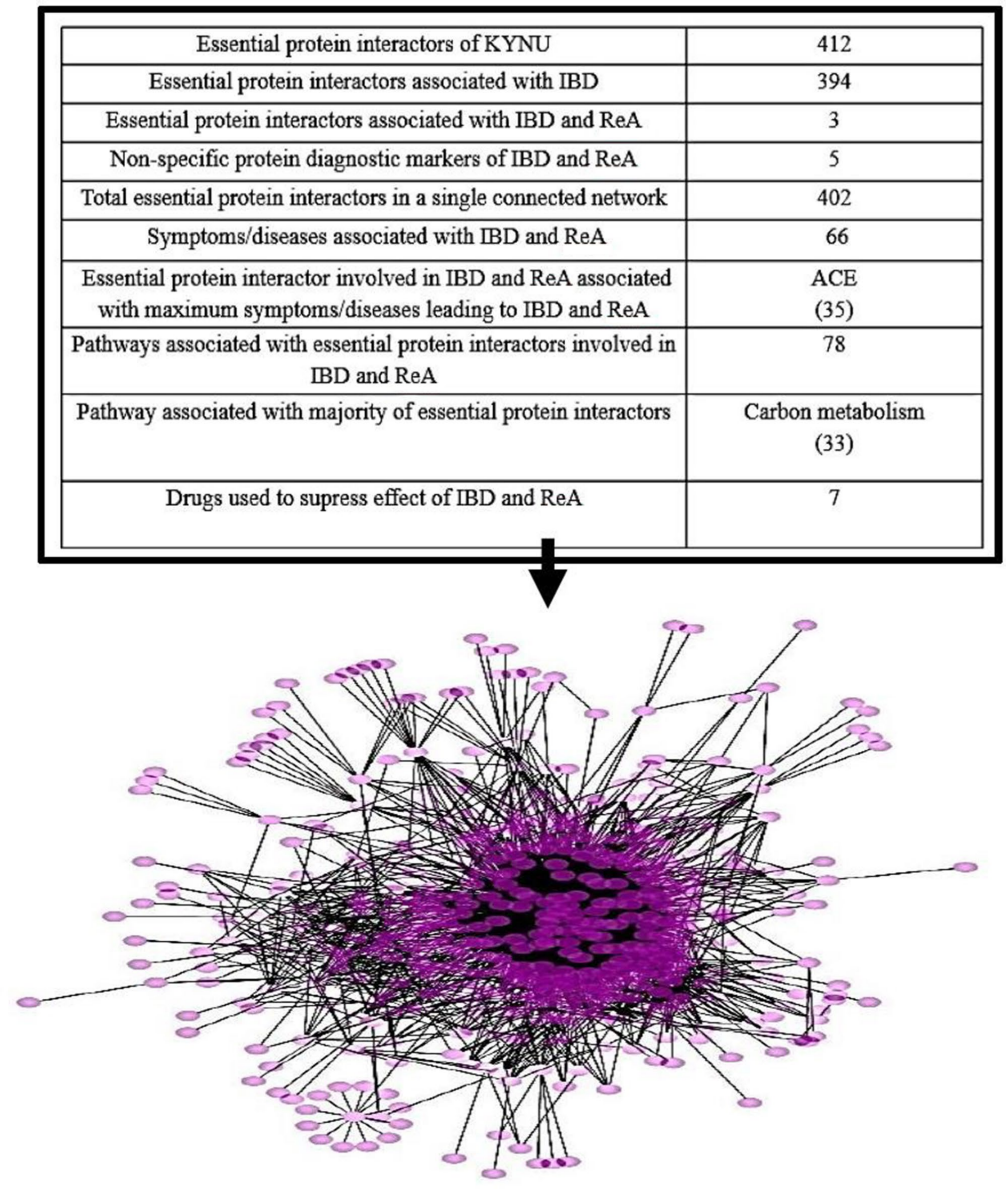
Host-microbe disease network (nodes = 555; edges = 3,319) of IBD co-evolved ReA.

The extensive interaction pattern of NHAA with KYNU along with 396 proteins, 5 markers, 66 symptoms/diseases, 78 pathways and 7 drugs give rise to a host-microbe disease network of IBD co-existent ReA (Fig. 6 and see Supplementary Table S14 online).

The final league of information processed in this study design was to accommodate the concept of molecular mimicry between the essential host proteins and selected microorganisms.

**Figure 7:**
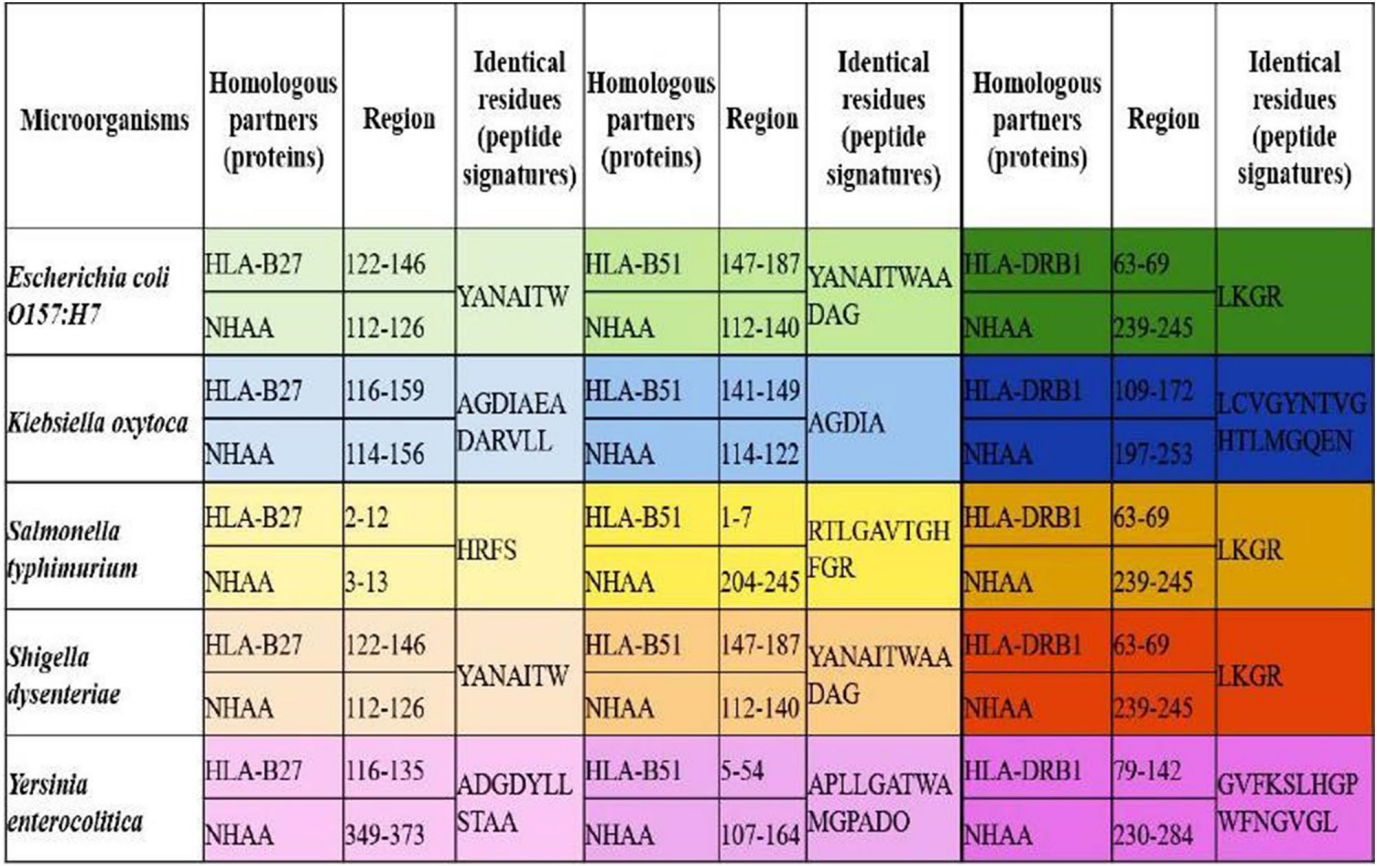
NHAA peptides from target microorganisms showing molecular mimicry with human HLA-B27, HLA-B51 and HLA-DRB1.

NHAA protein of target microorganisms shows homology with human HLA-B27, HLA-B51 and HLA-DRB1 (Fig. 7).

Peptides homologous to HLA-B27:

*Escherichia coli O157:H7* (112–126), *Klebsiella oxytoca* (114–156), *Salmonella typhimurium* (3–13), *Shigella dysenteriae* (112–126) and *Yersinia enterocolitica* (349–373).

Peptides homologous to HLA-B51:

*Escherichia coli O157:H7* (112–140), *Klebsiella oxytoca* (114–122), *Salmonella typhimurium* (204–245), *Shigella dysenteriae* (112–140) and *Yersinia enterocolitica* (107–164).

Peptides homologous to HLA-DRB1:

*Escherichia coli O157:H7* (239–245), *Klebsiella oxytoca* (197–253), *Salmonella typhimurium* (239–245), *Shigella dysenteriae* (239–245) and *Yersinia enterocolitica* (230–284).

### Experimental evidences to identify the signature molecules in patients

The in-silico analysis followed for the molecular signature identification till far through gene expression datasets and curated metabolic reconstructs strongly indicate the host protein, KYNU being the singular common predictive markers for all pathogenic microbes. KYNU has also been indicated in the expression data of inflammatory linked disorder, IBD. There is lack of data available regarding KYNU differential expression in ReA, therefore the experimental evaluation of KYNU through targeted expression analysis in ReA patients was carried out. A non-probabilistic convenience sampling was followed for our single blind study.

This study encompassed 15 individuals: 60% male with mean age of 45.7 and 40% female with mean age of 38 (9 males and 6 females). Out of these cases were: 10 with ReA and controls were: 3 currently undergoing treatment, 1 with Poncet’s Disease (PD) and 1 Healthy control (HC). The clinical characteristics of the patients recruited in the study included inflammatory back pain in 33%, fatigue in 60%, fever in 27%, swollen joint in 47%, Ankylosing Spondylitis (AS) that affects spine in 7%, dactylitis that is inflammation in finger or toe in 7% and Poncet’s Disease (PD) in 7% of participants. The clinical characteristics of the recruits are provided in Table 3.

**Table 3:** Sociodemographic and clinical characteristics of ReA cases vs controls.

Sample ID	Age	Gender	Clinical presentation	C-reactive protein (mg/L)	HLA-B27	Currently undergoing treatment	Duration of disease
1 (case)	34	F	Inflammatory back pain, fatigue, fever, swollen joint	30.60	N/A	NO	N/A
2 (case)	26	M	Swollen joint, diarrhea, gastrointestinal symptoms	61.4	N/A	NO	N/A
3 (case)	28	F	Ankylosing spondylitis, diarrhea	14	+	NO	N/A
4 (case)	65	M	Inflammatory back pain, fatigue, swollen joint	32.8	N/A	NO	N/A
5 (case)	44	M	Dactylitis, diarrhea, fatigue, swollen joint	56.5	N/A	NO	N/A
6 (case)	32	M	Swollen joint	25.1	N/A	NO	N/A
7 (case)	31	F	Fatigue, fever	1.4	N/A	NO	N/A
8 (case)	30	M	Fatigue, fever, swollen joint	47	+	NO	N/A
9 (case)	25	M	Fatigue	3.9	N/A	NO	7 months
10 (case)	50	F	Inflammatory back pain, fatigue, swollen joint	5.3	N/A	NO	N/A
11 (control)	29	F	Fatigue, fever, Poncet's disease	29.9	N/A	NO	N/A
12 (control)	56	F	Healthy	N/A	N/A	NO	N/A
13 (control)	57	M	Diarrhea, inflammatory back pain, fatigue	5.3	N/A	YES	N/A
14 (control)	60	M	Diarrhea	42.1	N/A	YES	N/A
15 (control)	73	M	Inflammatory back pain	27.80	N/A	YES	N/A

CRP normal levels (0–3).

N/A—data not available.

The expression of KYNU in Peripheral Blood Mononuclear Cells (PBMC’S) of ReA cases vs controls was evaluated using Relative Expression Software Tool (REST) software that estimated a sample’s relative expression ratio in relation to the control housekeeping gene (here GAPDH) by calculating an intermediate absolute concentration value:

$${\text{Concentration}} = {\text{efficiency}}^{{{\text{average}}\,{\text{CP}}\,({\text{controls}}) - {\text{average}}\,{\text{CP}}\,({\text{samples}})}}$$

where CP = point at which fluorescence escalates considerably above the background fluorescence.

Here the CP values for reference and target genes are collectively redistributed to control and sample groups and the expression ratios are calculated based on the mean value.

$${\text{Relative}}\,{\text{expression}} = {\text{Concentration}}\,{\text{of}}\,{\text{gene}}\,{\text{of}}\,{\text{interest}}/{\text{Concentration}}\,{\text{of}}\,{\text{reference}}\,{\text{gene}}$$

A Pair Wise Fixed Reallocation Randomisation Test is followed for normalisation of the target genes with a reference gene and for calculating the statistical difference of variation between 2 groups^81^. It utilises a bootstrapping technique providing a 95% confidence interval for expression ratios. It uses a P(H1) test for testing the significance between the samples and controls.

**Figure 8:**
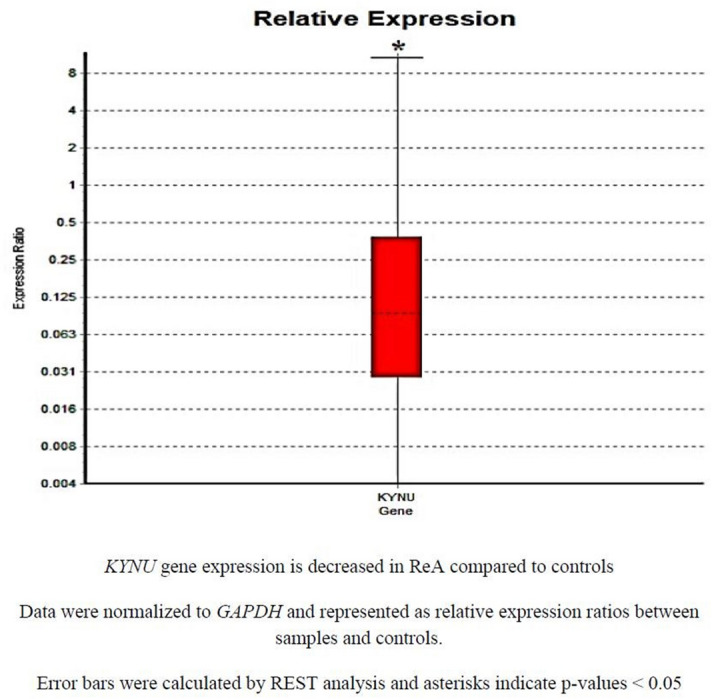
Relative expression of KYNU in cases vs controls.

According to our analysis, KYNU sample group is different to control group where P(H1) = 0.025. KYNU was found to be downregulated in sample group (in comparison to control group) by a mean factor of 0.115 (Standard error range is 0.018–0.837) as depicted in the whisker-box plot (Fig. 8). KYNU expression showed a ~ ninefold decline in ReA cases as compared to controls.

## Discussion

Gut microbiome is pitched to be the central theme housing enormous diversity of microbial species, characterizing the fine balance between healthy and diseased states. The physiological drifts from healthy to diseased and vice-versa is tuned to sophisticated interactive networks of human host and the microbial flora residing the gut. The autoimmune conditions Reactive Arthritis (ReA) and Inflammatory Bowel Disease (IBD) have been linked to prevalent dysbiosis of the gut, where disease development occurs as a perceptive reaction due invading population of microbes. To find out the basal networks of interactions at the host-microbe interface, common microbes affecting the co-evolved diseases with shared characteristics were studied. These involved comprehensive analysis of the bimolecular functional networks including the gene, protein, metabolite molecular signatures engraved at the host-microbe and microbe- microbe interface. This ‘interspecies communication’ have been linked now with immuno-pathogenesis of most human autoimmune disorders^82,83^.

The etiopathology of these interactions have remained elusive leading to non-specific diagnostic criteria and therapeutic regimes. It is suggested that microbial dysbiosis, pathogenic infection and host-microbe interactions cause incidence of ReA. In this study, utilising the combinatorial approach we have compiled a repertoire of microorganisms, biomolecules and pathways that are possibly involved in triggering co-evolved autoimmune disorders IBD and ReA. In our study, text mining results convey the presence of microorganisms namely *Campylobacter jejuni*, *Escherichia coli O157:H7*, *Klebsiella oxytoca*, *Salmonella typhimurium*, *Shigella dysenteriae and Yersinia enterocolitica* implicated in both the disorders.

The thematic concepts for microbe contribution in host immunity have been explored in our previous analysis of metabolic reconstruction and simulation of *Campylobacter jejuni* and *Salmonella enterica*^69,84^. In our current study, we used a designated work-pipeline for metabolic network reconstruction and simulation of target microorganisms. The analysis conducted extracted the information via constraint-based bottom-up approach that was filtered and utilised for further computational analysis. The essential genes, proteins and metabolites of microorganisms represent the promising drug targets as these are speculated to contribute towards infection triggered host physiological drifts leading to development of the co-evolved pattern of autoimmunity in IBD and ReA.

A thorough curation pattern followed led to provide robust molecular cues in terms of essential proteins and biological networks that are correlated to the ‘interspecies communication’ using the host-microbe and microbe-microbe interaction profiling. The most closely associated common protein observed in all the selected common microbial species involved in both IBD and ReA is Na^(+)^/H^(+)^ antiporter (NHAA), microbial integral membrane protein, catalyzing the exchange of 2 H^(+)^ per Na^(+)^^85^ and involved in processes crucial for cell viability.

Similarly, the common host interacting protein with NHAA is Kynureninase (KYNU), involved in tryptophan metabolism and whose differential expression (upregulation and downregulation based on the control samples) have been followed in IBD patient cohorts^86–88^. As per the scientific discourse presented in the studied disorders, the pathological mechanism hypothesizes that after bacterial infection, antigen-presenting cells transport bacterial antigens/peptides into the synovial membrane, where the bacterial components persist causing inflammation. It is suggested that in host-microbe interactions, bacterial proteins entering host cells interact with host proteins and inject their effector components, but has not been proven in ReA and IBD. So, this formed a basis of one of the parameters in our study design where we found the physical interactions between NHAA and KYNU and predicted that these might be the early host-microbe interactors for establishing pathogenesis in IBD associated ReA.

This could assist to comprehend the very few reports indicated in the rare autoimmune ReA, where gene expression datasets of the co-evolved disorder IBD can serve to incorporate the larger theme of gut-microbiome associations. The theme of gut-microbiome paradigm shifts thus contemplates the vital cues in triggering autoimmunity with indirect linkages to diet and environmental triggers. This is indicative of the identified target molecular signature, KYNU, found to be differentially regulated in the patient cohorts with history of infection triggered or IBD co-evolved ReA. KYNU and NHAA could serve as the robust early and essential host-microbe interacting targets and molecular indicators involved in interspecies communication in IBD associated ReA.

The investigations further were targeted for parallel analysis of other host-essential protein partners enmeshed to have interaction with host protein KYNU indicating the intricate details of host-microbe interaction information. The disease network constructed through our approach consists of 412 single connected essential protein interactors of KYNU, where 394 human essential protein interactors are found to be associated with IBD, while 3 of them (Adenosine Deaminase (ADA), Catalase (CAT) and Superoxide Dismutase 2 (SOD2)) are associated with both IBD and ReA. ADA protein has been reported in Juvenile Idiopathic Arthritis and ReA patient cohorts in serum samples^89^. Similarly, CAT and manganese superoxide dismutase (SOD) genes polymorphisms were observed in ReA patient cohorts^90,91^. These become part of the host-microbe disease network where such molecular elements and co-regulatory pathways represent the intricate biological cross-talk followed during disease development.

Pathological conditions can also trigger immune cells such as IL’s and TLR’s and various cytokines leading to immune cell infiltration in host and higher levels of inflammation. Genetic factors such as HLA alleles encode susceptibility, contribute to bacterial persistence and increase risk in ReA cases. Based on this we also found the interactions of important targets in our study with immunogenic and genetic factors. The host harboured assorted essential proteins were further probed for their association with non-specific protein diagnostic markers as well as with symptoms/diseases linked with IBD and ReA, accruing towards a single connected network consisting of 402 interdependent proteins.

The reciprocation of these integrated protein indicators to the disease development is conveyed through metabolite monitoring as in the study, Angiotensin I Converting Enzyme (ACE) was found to be linked with maximum symptoms/diseases.

ACE is involved in catalyzing the conversion of angiotensin I into angiotensin II that is a potent vasopressor and aldosterone-stimulating peptide that controls blood pressure and fluid-electrolyte balance^92^. This could be the indicator of involvement of microbe triggered host physiological drifts. Subsequently, the pathways associated with the proteins ramified into 78 pathways of human host speculated to give details of metabolic regulatory checkpoints where carbon metabolism is found to be associated with majority of deduced proteins. Carbon metabolism pathway implicated here as the vitally generic pathway for IBD co-related ReA confers how diet, balance of gut microbiome, antibiotic exposures can have layered impact on autoimmune disease progression and remissions. KYNU is found to be downregulated in ReA patients as compared to controls through our targeted gene expression analysis.

Collectively, the disease network followed here confers interaction of microbial NHAA with host KYNU, that is further correlated to 396 proteins, 5 markers, 66 symptoms/diseases, 78 pathways and 7 drugs. Docking analysis of drugs used to suppress the effect of IBD and ReA predicts methotrexate as an important drug that could be useful for early treatment of IBD co-evolved ReA.

Genetic factors found common in both ReA and IBD are HLA-B27, HLA-B51 and HLA-DRB1. The most important mechanism of susceptibility of HLA in ReA is molecular mimicry that is microbial peptides mimicking HLA autopeptides of human host leading to autoimmunity. This mechanism has been observed in ReA where reports have predicted microorganism peptides such as chlamydial proteins (ClpC, NQRA and DNAP) and *Yersinia pseudotuberculosis *peptides (YopH) showing homology with human HLA-B27 via bioinformatic analysis^14^. Similarly, molecular mimicry has also been observed in IBD cases having extraintestinal manifestations. We performed targeted molecular mimicry analysis in our study using our robust microbial protein (NHAA) with HLA-B27, HLA-B51 and HLA-DRB1, enhancing the importance of NHAA acting as a trigger for generating IBD associated ReA.

We generate a putative hypothesis amalgamating key findings with literature. We state that the initial host-microbe triggers for IBD associated ReA is when pathogenic microbial protein NHAA interacts with host protein KYNU that further interacts with human proteins ADA, SOD2, CAT and ACE and carbon metabolism involving the above host proteins is hampered. Methotrexate regulates carbon metabolism and the associated host-microbe proteins reducing effect of IBD associated ReA.

Since carbon metabolism is the most basic aspect of life and therefore an extensive network consisting of sub-pathways, we narrowed down our findings towards a consequentially central and a significant pathway that embrace the carbon metabolism pathway involving the molecular signatures KYNU, ADA, SOD2, CAT and ACE, further is also effectuated by potential drug methotrexate and is associated with IBD/ ReA/ IBD and ReA cohorts.

It is reported that methotrexate is incorporated intracellularly interfering with adenosine concentrations and affecting proinflammatory cytokines in IBD reducing inflammation^93^. In inflammatory arthritis, the mechanisms reported by which methotrexate reduces inflammation include enhanced adenosine release, de novo synthesis of purines and pyrimidines, inhibition of transmethylation reactions, diminished accumulation of polyamines and nitric oxide synthase uncoupling. Most of the mechanisms are associated with folate biosynthesis, a type of carbon metabolism^94^. KYNU, ADA, SOD2, CAT and ACE are also found to be involved in folate biosynthesis and metabolism from GeneCards.

Apart from the above targets, parallel interactors, pathways and drugs for IBD co-evolved ReA obtained in our host-microbe disease network can be utilised further as disease determinants. The experimental validation of these targets in patient cohorts need to be performed on a pilot scale in future to increase the robustness of this network.

The intertwined information processed through the knowledge-base created for the linked disorders have given the most elaborate layout of patterns observed in disease diagnosis and analysis. The major information after processing the gene expression profiles, protein markers, molecular networks and metabolic networks involved here have led to chalk out as well as connect the strings for robust gut microbiome paradigm shifts.

## Conclusions

The current work on host-microbe interactions provides a starting point for researchers and clinicians to investigate Inflammatory Bowel Disease (IBD) associated Reactive Arthritis (ReA). In this study a combinatorial approach is utilised to reveal the interactions of gut microbes with human host extensively sketched through the work-pipeline providing the vital insights for the drug targets, biomarkers, pathways and inhibitors for etiology, prognosis, diagnosis and treatment attributes of pathogenic rheumatic autoimmunity.

The information sorted through the combinatorial study will be useful in deciphering the etiopathogenesis of the co-linked disorders especially for the rare ReA, from synonymous analyses of IBD datasets, conferred through common microbial triggers.

These predictions substantially furnish the intricate details of the cross-talk between post-infectious inflammatory reactions with shared patho-immunogenesis as the starting point for researchers and clinicians for detailed and newer experimental analysis. Future studies are required on larger cohort of patients having ReA due to IBD in order to have validated outputs of the predictive network.

## Supplementary information

Supplementary Table S1.

Supplementary Table S2.

Supplementary Table S3.

Supplementary Table S4.

Supplementary Table S5.

Supplementary Table S6.

Supplementary Table S7.

Supplementary Table S8.

Supplementary Table S9.

Supplementary Table S10.

Supplementary Table S11.

Supplementary Table S12.

Supplementary Table S13.

Supplementary Table S14.
